# Predicting choice behaviour in economic games using gaze data encoded as scanpath images

**DOI:** 10.1038/s41598-023-31536-5

**Published:** 2023-03-23

**Authors:** Sean Anthony Byrne, Adam Peter Frederick Reynolds, Carolina Biliotti, Falco J. Bargagli-Stoffi, Luca Polonio, Massimo Riccaboni

**Affiliations:** 1 MoMiLab Research Unit, IMT School for Advanced Studies Lucca, Lucca, Italy; 2 AXES Research Unit, IMT School for Advanced Studies Lucca, Lucca, Italy; 3 Department of Biostatistics, Harvard University, Boston, USA; 4 Department of Economics, Management and Statistics, University of Milano - Bicocca, Milan, Italy

**Keywords:** Human behaviour, Data mining, Socioeconomic scenarios, Computational science

## Abstract

Eye movement data has been extensively utilized by researchers interested in studying decision-making within the strategic setting of economic games. In this paper, we demonstrate that both deep learning and support vector machine classification methods are able to accurately identify participants’ decision strategies before they commit to action while playing games. Our approach focuses on creating scanpath images that best capture the dynamics of a participant’s gaze behaviour in a way that is meaningful for predictions to the machine learning models. Our results demonstrate a higher classification accuracy by 18% points compared to a baseline logistic regression model, which is traditionally used to analyse gaze data recorded during economic games. In a broader context, we aim to illustrate the potential for eye-tracking data to create information asymmetries in strategic environments in favour of those who collect and process the data. These information asymmetries could become especially relevant as eye-tracking is expected to become more widespread in user applications, with the seemingly imminent mass adoption of virtual reality systems and the development of devices with the ability to record eye movement outside of a laboratory setting.

## Introduction

The use of eye-tracking as a means of information acquisition for automated systems is ever-increasing due to technological advances such as the ability of front-facing cameras on smartphones^1,2^ and laptops^3,4^ to accurately record eye-movements at scale. Other developments, including the growing presence of virtual and augmented reality devices in everyday life^5^, have also contributed to the development of eye-tracking software and devices. Further, recent advances in Machine Learning (ML) techniques have led to a significant increase in the accuracy of prediction when modelling gaze data^1,6–8^. ML techniques have been applied to eye-tracking data to model human cognition in a variety of settings, including – but not limited to – detecting sarcasm^9^, identifying when a participant is in a state of confusion^7^, classifying the relevance of a passage text to a user^10^, and predicting where a participant will focus their attention during location-based games^11^. Further, humans are more frequently interacting with automated systems when engaging in strategic contexts, which is a phenomenon that has been noticed by policy makers^12,13^. Taken together, these factors seem to suggest that gaze data may soon be incorporated into online automated systems that will be able to anticipate future decisions of a user based on their gaze behaviour.

Numerous studies have demonstrated that gaze patterns can be effectively used to model the decision-making process of individuals operating in strategic contexts (e.g., economic games), which can, in turn, be used to form a prediction regarding if they will choose an optimal strategy or not^14–16^. As eye-tracking becomes more widespread in user applications, it follows that an Artificial Intelligence (AI) system will be able to incorporate gaze data in order to anticipate the choices of a human agent across a broader range of strategic contexts introducing an information asymmetry in favor of the automated system and those who control the technology. The onset of gaze-aware systems may bring many potential benefits to the user. An example of this benefit is providing a means to create early detection systems which can be used to warn users before they make sub-optimal choices or to provide, in training scenarios, adaptive feedback based on the analysis of the users’ pattern of eye-movements^8,17,18^.

Under the tightly controlled environment of economic games, we explore a method of anticipating the decision strategy of an individual by modelling the pattern of their eye movements. We analyze gaze data recorded during an experiment where participants play a set of games commonly used in the field of economics to study interactions in strategic settings (see ”The Games” subsection for a complete description). Participants play the games on a computer screen against an algorithm that simulates the behavior of a rational player who aims to maximize their profit by choosing the Nash equilibrium strategy, which is the optimal strategy under the assumption that the counterpart is also rational and profit-oriented. The participants are informed that the algorithm is rational and profit-oriented before the game starts. Therefore any deviation from the equilibrium solution, which assumes rationality and profit maximization from both agents, can be exclusively attributed to the participant’s inability to identify the optimal strategy^19^. In this setting, deviations from equilibrium can be systematically categorised as different types of sub-optimal strategies. To model the decision-making process associated with different decision strategies, we create images that express gaze patterns. These images are generated from raw eye-tracking data, which capture the temporal sequences of eye fixations and are commonly referred to as *scanpaths*^20,21^. We consider each scanpath image as a viable proxy measurement for the temporal evolution of the decision process with which participants acquire and integrate information.

**Figure 1:**
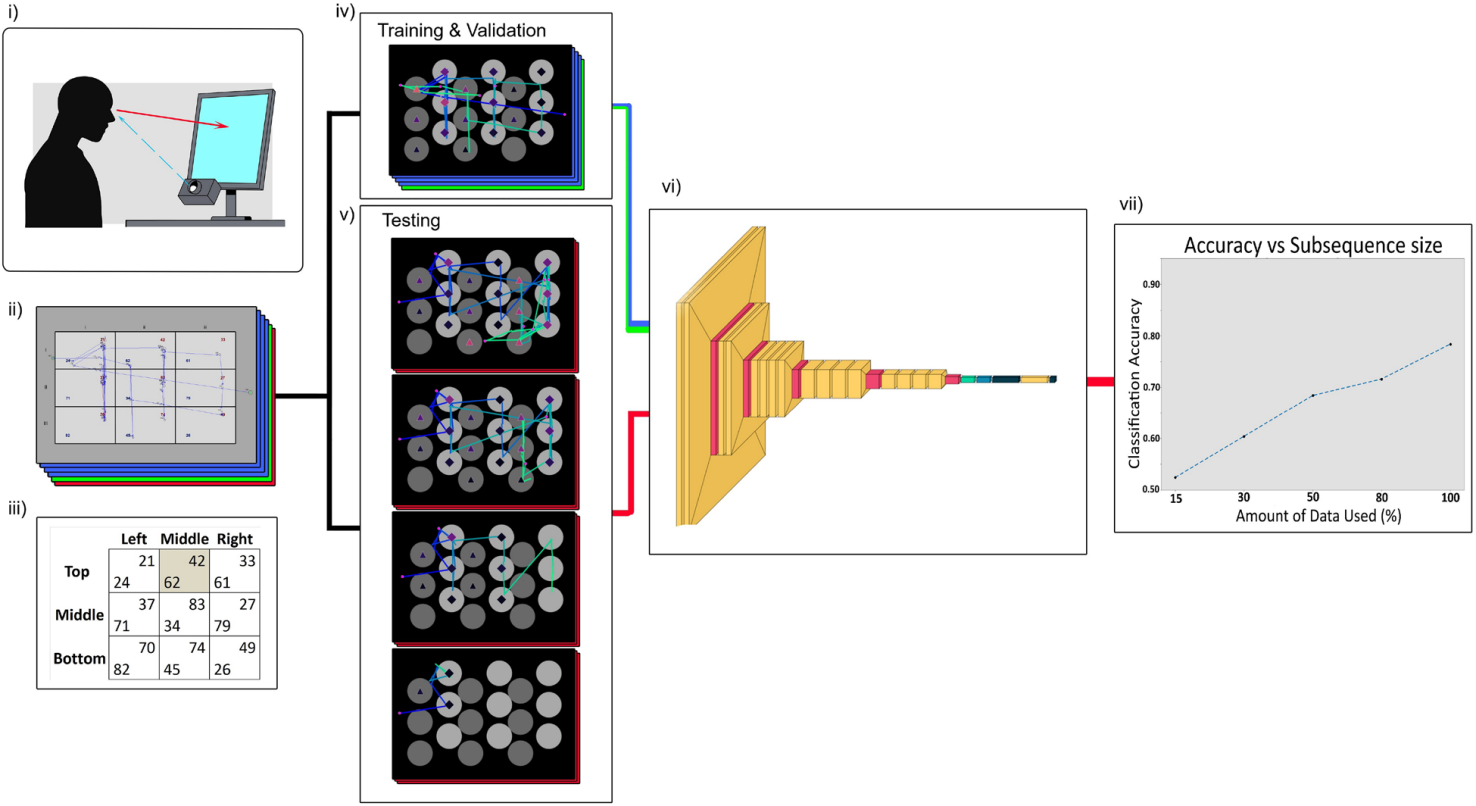
Overview of approach: (**i**) In this eye-tracking study, the gaze behavior of the participants was recorded as they played games presented on a computer screen, using a tower mount eye-link with a sample rate of 1000 HZ (**ii**) An example of how we presented the games during the eye-tracking experiment. The payoffs of the Row player are coloured in blue and the payoffs of the Column player are in red. The payoffs are separated by the maximum distance allowing for the clearest possible distinction between ocular events that happened in different areas of interest. In this example, the raw gaze recording of a single participant is displayed on the game board in order to highlight the difference between the raw gaze data and the scanapath images used as model input (**iii**) An example of how a two-player strategic interaction can be represented using games presented in normal-form. The Row player (the human participant in our experimental task) can choose between the actions “Top”, “Middle” and “Bottom”. The Column player (the algorithm in our experimental task) can choose between the actions “Left”, “Middle”, and “Right”. The action selected by the Row player affects the payoff received by the Column player and vice-versa. The game’s outcome is the cell given by the intersection between the action selected by the Row player and the action selected by the Column player. The Row player receives the payoff located in the bottom-left part of the cell, and the Column player gets the payoff located in the upper-right part. The equilibrium of the game is highlighted in gray. (**iv**) Before performing any analysis, we split the data into three sets of participants. We allocated approximately 70% of the participants for training the model, 20% for validation of the results, and kept 10% of the participants as a hold-out test set. (**v**) Using the hold-out test, we consider a series of independent model tests where we create sets of shortened scanpaths based on criteria such as percentage, or only allowing scanpaths to be created within a certain amount of time (e.g., 2 s or 5 s). (**vi**) We pass these sets of scanpaths through our fully trained model, testing its predictive ability. (**vii**) A stylised graphical representation of our findings, we used model accuracy as the main metric to compare the results and noticed only a small decrease in accuracy relative to the amount of data we removed from the scanpath.

We use the scanpath images in two machine learning Classification Tasks (CTs). In CT 1, we set up a simple binary classification task aimed at determining if the participant chooses the equilibrium strategy or not. In CT 2, we classify the exact strategy of the participant from the three available options in the game (see Results Section for a detailed description of how these options relate to strategic profiles). To demonstrate a clear example of how this method can produce an information asymmetry, we expand our analysis by running a series of machine learning experiments after each CT by using the trained models to classify scanpaths created from only a subsequence of the available gaze data starting from the beginning of the recording (see Fig. 1 for an overview of our approach). The results of these experiments provide evidence that the gaze patterns of participants who chose the equilibrium action, as well as actions consistent with different strategic profiles, are detectable with very little gaze data and critical to our hypothesis, using data recorded before the participant committed to a choice in the game. This, in principle, would allow an algorithm capable of processing this information in real-time to anticipate the future choice of a player. We achieve these results by predicting the choices of participants unseen by the model during training and validation since the model has only been trained using scanpaths generated from full sequences. Using a modeling method that only requires gaze data, we provide evidence that the decision strategy of an individual is detectable independently of the specific strategic structure of the game under consideration. Moreover, we show that our approach in principle would be able to predict the decision strategy of an individual also in strategic environments that were never seen by the algorithm.

Many different classification approaches have been previously applied to gaze patterns in order to model the decision-making process of individuals and classify their decision strategy in economic games^14–16,19,22,23^. We contribute to this literature by investigating if presenting the data as a scanpath image, and thus accounting for the spatiotemporal patterns of the data, provides any additional benefits to increasing the accuracy of prediction. More generally, significant work has gone into creating systems with the capability of adaptive feedback using gaze data as model input. Notable work includes distinguishing between novice and expert dentists^8,24^, a tool for training radiologists^17^, and detecting drivers’ state while operating a vehicle^25,26^. We contribute to this literature concerning human scanpath classification by demonstrating that in some environments, scanpaths generated from subsequences, or in other words partial scanpaths, are sufficient for a model trained on full scanpaths to classify a user’s strategy in complex strategic contexts.

Our study has three objectives. First, we aim to present a new method to analyse eye patterns recorded during economic game playing with a higher classification accuracy when compared to traditional methods. Second, we intend to provide a clear example of gaze data being used to create an information asymmetry in favour of those that in the future might develop this technology in real-time scenarios. From a policy perspective, we hope to highlight the clear need for regulation surrounding how gaze data and, more generally, biometric data are processed and used as technology becomes rapidly more immersive, making this type of data more available to AI systems. Third, we aim to present a method of early detection via the creation of scanpath images from subsequences of the data, which may be useful to the more general literature focused on creating an adaptive online system that incorporates eye-tracking data.

## Results

### Models of choice and behavioral results

The games used in this study are characterized by a unique game theoretical optimal solution known as the Nash Equilibrium^27^. We used data from participants playing with an algorithm that always selects the equilibrium strategy. At the beginning of the experiment, participants are informed that the algorithm will play rationally by trying to maximize its own payoff and that it cannot modify its strategy during the experiment nor adjust its choices to those of the human player. In all games, equilibrium play requires the participant to use strategic sophistication. Strategic sophistication is defined as an attempt to predict the behavior of the counterpart by taking its incentives into account^28^. Equilibrium play in this type of games is commonly associated with an information search pattern focused on evaluating the incentives of the counterpart, detection of the possible presence of dominant strategies, and identification of the other player’s action with the highest average payoff. Deviations from equilibrium are consistent with decision rules such as Naïve and Coordination strategies. A Naïve strategy predicts if the participant selects the action with the highest average payoff. This strategy is commonly associated with a search pattern focused on the payoffs of the participant while the payoffs of the counterpart are barely considered. A Coordination strategy predicts if the participant selects the action consistent with the outcome that yields the largest payoff sum for the two players minimizing the difference between the two payoffs. This strategy requires the participant to compare the payoffs of the two players for each possible outcome of the game^16,19,23,29–31^.

Looking at the entire pool of data (243 participants, 2 430 choices) the proportion of choices consistent with the Equilibrium strategy is equal to 0.48, whereas the proportion of choices consistent with Naïve and Coordination strategies is equal to 0.31 and 0.21, respectively. The average response time is 15 980 ms (SD = 13 320 ms) but this value changes significantly based on the strategy used by the participant. Equilibrium choices (18 050 ms, SD = 13 200 ms) take longer than choices consistent with Naïve (13 770 ms, SD = 16 780 ms) and Coordination (15 660 ms, SD = 15 400 ms) strategies. This is supported by the results of a mixed-effects logistic regression with Equilibrium Response (1, 0) as the dependent variable, logarithmic transformation of the Response Time data as the independent variable, and Subject as Random effect (B = 0.38, p< 0.001). This difference in the average response time is well documented and reflect the different level of complexity of the three strategies. Response times are not affected by the game type: on average, participants take 15 840 ms (SD = 13 380 ms) to make a decision in games that are solvable through iterated dominance and 16 120 ms (SD = 13 840 ms) in games that are not.

In terms of search patterns, equilibrium responses are characterized by a high proportion of transitions (defined as eye movements from one payoff to the next) among the payoffs of the counterpart (0.29) and a lower proportion of transitions among the payoffs of the participant (0.18). Choices consistent with the Naïve strategy are characterized by a higher proportion of transitions among the participant’s payoffs (0.36) and a lower amount of transitions among the payoffs of the counterpart (0.11). Finally, choices consistent with the Coordination strategy are associated with a more balanced proportion of the two types of transitions (participant = 0.26; counterpart =0.22). The analysis of fixation times yields results that are identical to those obtained with transitions: the average amount of time participants spend looking at the payoffs of the counterpart is longer than the amount of time they spend looking at their own payoffs when they play equilibrium (own =5 516 ms; other = 7 695 ms), shorter when they play the Naïve strategy (participant =5 996 ms; counterpart = 3 549 ms), and almost identical when they play the Coordination strategy (participant = 5 822 ms; counterpart = 5 520 ms).

### Modelling cognition in games using scanpaths

**Figure 2:**
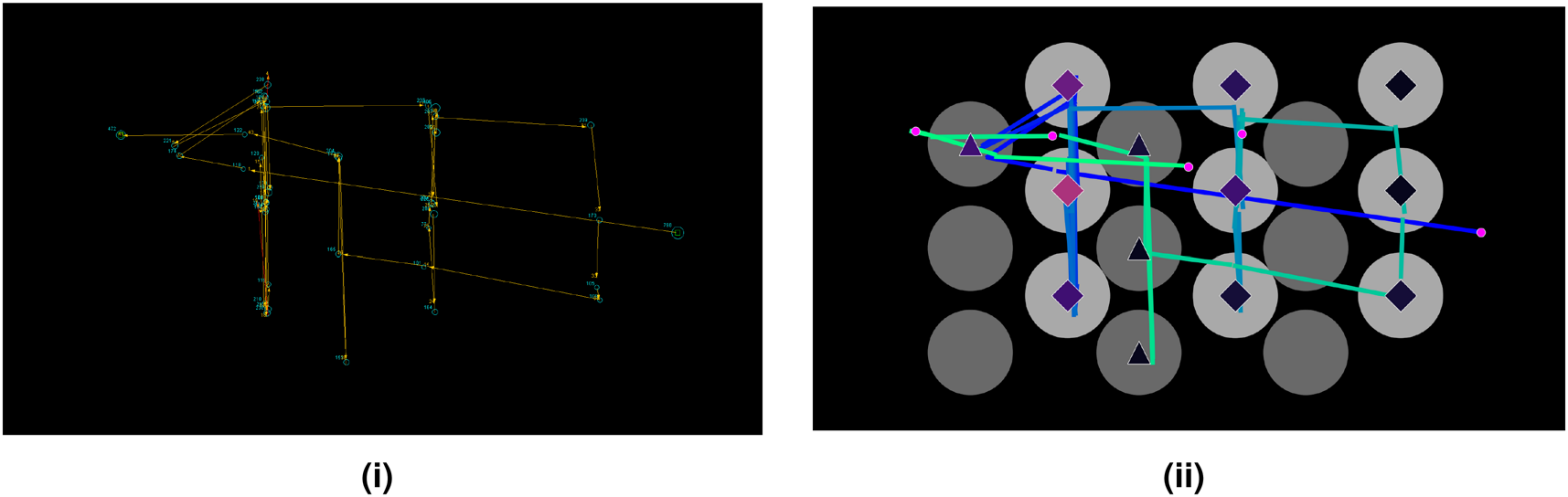
(**i**) A prototypical representation of a scanpath displaying fixation locations, fixation duration, and saccades. (**ii**) An example of how we represent the data using scanpaths to increase the salience of information to the models. The circles represent the location of the payoffs for both the participant (light grey) and the opponent (dark grey). We use sequential colourmaps to represent the temporal evolution of the linear saccades and to display information regarding fixations to the model.

To untangle the participants’ decision-making process we first transform the raw eye-tracking data into scanpath images. We use the following techniques to increase the salience of features that are predictive of choice behavior in the scanpath images. These features include the temporal evolution of the visual analysis and the number of times a particular piece of information is acquired. We first define 18 Areas of interest (AOIs) centered on the matrix payoffs and distinguish between fixations that happened inside or outside of these areas. AOIs centered on player’s own payoffs (payoffs the participant could receive) are light-gray circles and AOIs centered on the counterpart’s payoffs are dark-gray circles. See Fig. 2 for an illustration of a standard representation of eye-tracking data and how we represent recorded data into a scanpath.

When creating the scanpaths a black background was used with the AOIs superimposed on top. We provide no information regarding the game environment other than the spatial coordinates of the AOIs, as this method allows us to isolate the relationship between the information acquisition process of a player and the resulting choice without taking into account any feature of the game structure. This is important because often the last fixations of a player fall on the expected outcome for the player, or on the label of the chosen option. We believe that this would be the primary source of information used by the model to make its prediction. Therefore, we hypothesized that providing information relating to the last fixation made on the game matrix would facilitate the prediction of the model when an image with the full visual pattern is available. However, when the images are generated from subsequences, where the last fixations are missing, this task would be much more difficult.

**Figure 3:**
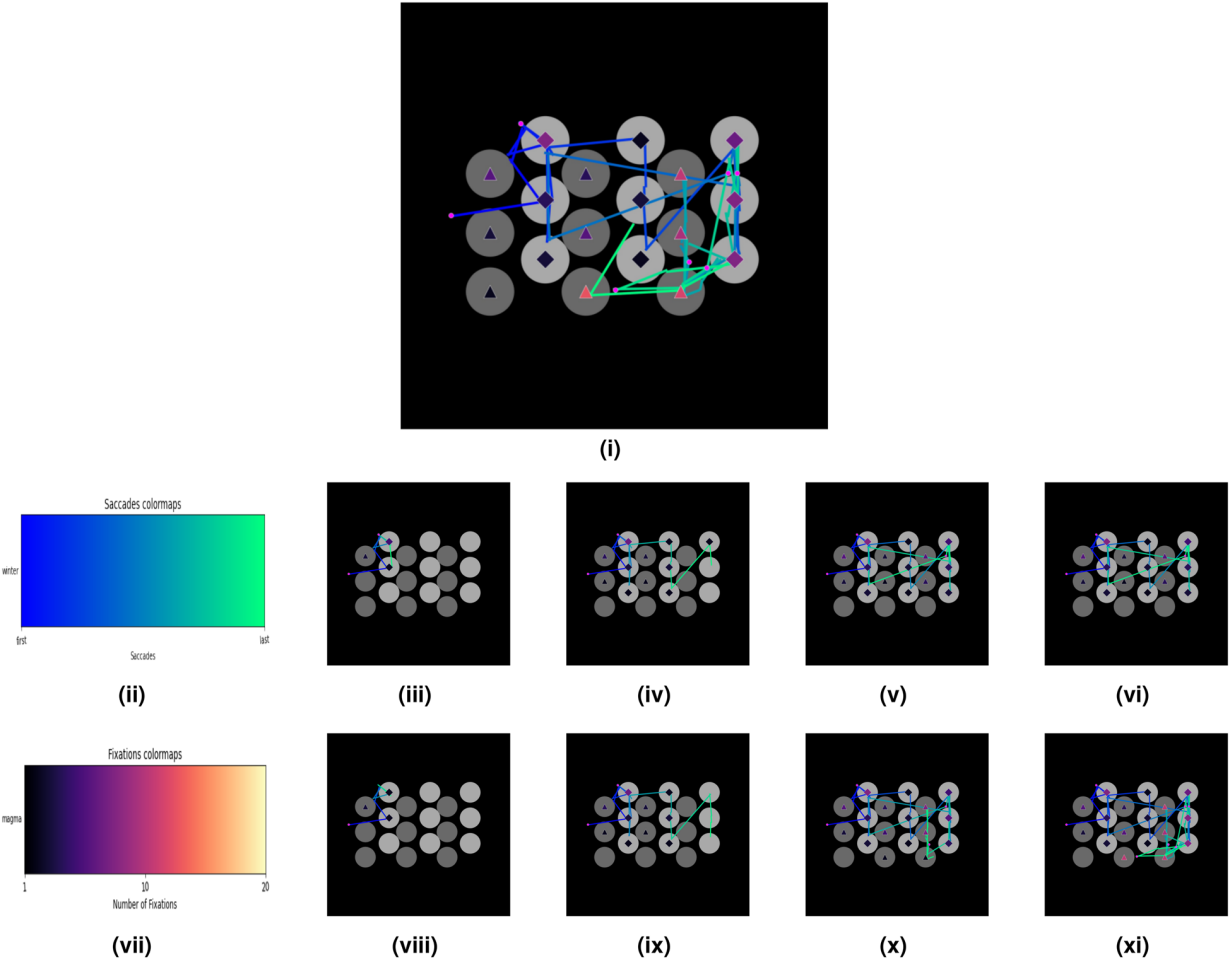
Scanpaths generated from the full sequence and subsequences of the data from one participant in a single game. In total, there are 8 sets of test scanpaths made subsequences. (**i**) Example of a full image, (**ii**) The colour-map used for saccades. The left side would correspond to colours of earlier saccades with the right side corresponding to later saccades. (**iii**, **iv**, **v**, **vi**) Images generated from subsequences stemming from the same participant-game at increasing percentage intervals. (**vii**) The colourmap chosen for fixations. The upward threshold of 20 fixations was chosen because 99% of the area of interest across trials across participants had 20 fixations or less. (**viii**, **ix**, **x**, **xi**) Images generated from subsequences stemming from the same participant-game at increasing time intervals.

To prevent overcrowding in the scanpath design, a single shape located in the center of each AOI represents the fixations occurring within each AOI. We use a triangle for own payoff and a diamond for when the participant gazes at the counterpart’s payoff. We use changes in colour from a perceptually uniform sequential colour map to represent when multiple fixations occurred within an AOI; this means that the lightness value increases monotonically throughout the colourmaps^32^. For example, if there was a single fixation in an AOI, the colour of the fixation shape would be black, and if there were ten fixations in the area of interest, the colour of the shape would be pink, following the “Magma” colour map, as illustrated in Fig. 3vii. All fixations outside of the AOIs are represented by Fuchsia Dots that are 57% the size of the fixation shapes within an AOI. We use linear saccades to represent the transitions between consecutive fixations taking the direct linear distance between fixation points. To capture the temporal evolution of the decision process, we colour-code each saccade. Earlier saccades are coloured a dark blue, RGB (0, 0, 255), and later saccades are coloured a light green RGB (0, 1, 254.5) as illustrated in Fig. 3ii. The saccades were designed using start-screen position (pixel), end-screen position (pixel), start-time of the saccade (ms), and end-time of the saccade (ms).

To create the sets of partial scanpaths we use the following methods. First, we create scanpaths using a percentage of the total gaze at the following intervals 15%, 30%, 50% and 80%, See Fig. 3iii–vi for an example of how partial scanpaths at each percentage interval compares to the participant’s full scanpath as illustrated in Fig. 3i. This method ensures that all the test scanpaths are equally reduced but with the drawback of needing to be calculated post-hoc. Second, partial scanpaths were created based on the length of time (2s, 5s, 10s and 15s). While this method is a more realistic representation of an online setting, it comes with a disadvantage that the amount of time it takes each participant to play a single game varies. This problem leads to situations occurring, such as when a participant finishes in a given game within the first 8 seconds, the partial scanpaths in the 10 s and 15 s sets are identical and are indeed full scanpaths. This feature of creating partial scanpaths by the length of time makes an overall comparison between the experiments difficult. See Fig. 3viii–xi for an example of how partial scanpaths created at each recorded time interval for a given participant compares to the participant’s full scanpath as illustrated in Fig. 3i. The average time taken per trial was 16 s, with the median time per trial being 11 s. To minimise the situation where test scanpaths are identical across test sets, the timings were chosen to be below the average time taken per trial.

### The dataset

In total, we use data from 243 individuals, with each participant playing 10 games, leading to a total of 2 430 scanpaths which we use as input to the model. We do not discard any of the 2 430 scanpaths from our study. We implement an approximate 70-20-10 train-validate-test split at the participant level. Previous studies have shown that both the strategy and visual analysis used by the same participant are consistent in different games. We observed this consistency in our data (see Supplementary Table 4), therefore we decided to split the data at the participant level. Moreover, we hypothesized that scanpaths recorded from the same participant and game type could cause an accidental leakage of signal across the training and testing sets. For an even comparison, we use the same data split for each experiment across our analysis, as we hypothesized that some participants would create harder-to-classify scanpaths. 1 700 scanpaths were randomly placed into the training set, 480 scanpaths into the validation set, and 250 scanpaths were placed into the test set.

### Model selection, machine learning modelling tasks and performance metrics

After generating the scanpaths we use the resulting images as model input into a VGG-19 model pre-trained on the Imagenet dataset. VGG-19 is a variant of the VGG architecture of convolutional neural networks consisting of 16 convolutional layers and is a standard model architecture used in computer vision tasks due to excellent performance that was conducive to its success in the 2014 Imagenet Challenge^33^. The VGG-19 architecture was chosen as it is a relatively shallow network with multiple small kernels which we hypothesised would be optimal for capturing any nuanced differences between the input images. Our hypothesis stems from results in an earlier paper that tests similarly a scanpath design on a wide range of out-of-the-box neural network models during a reading task^10^. To further validate our preferred VGG-19 method, we also report the results of two benchmark cases: a Support Vector Machine (SVM) configured for image classification, commonly used in scanpath classification tasks and a logistic regression model which is the most common method of traditional analysis to test the link between gaze data and choice behavior in games presented in normal-form.

We configure all the models employed for two independent Machine Learning Classification Tasks (CTs). CT 1 is a binary classification task, where the models are tasked with detecting if a participant selects Nash-Equilibrium or not. CT 2 is a multi-class classification problem where the models are asked to identify the exact strategy profile of the participant (Naïve, Coordination, Nash-Equilibrium). Importantly, the equilibrium location change in different games and each decision strategy is independent of the three available actions (”Top”, ”Middle”, ”Bottom”). A full description of the games, the spatial location of the equilibrium and of the three different strategies are available in Fig. 2 of the Supplementary Material. A convenient feature of our dataset is that in CT 1 the class labels are almost naturally balanced, making it easier to train the model^34^. As an initial pre-processing step in CT 1, we create a fully balanced dataset by randomly under-sampling the majority class, removing a total of 46 scanpaths (corresponding to 1.89% of the data). In CT 2, the labels create an imbalanced dataset with almost double the responses in the majority class (Nash-Equilibrium) compared to the other two classes. This second CT allows us to test our approach on a small imbalanced dataset, which is more indicative of real-world problems^34–36^.

We assess model performance using a set of widely used accuracy measurements including Accuracy, Area Under the Curve (AUC), and F1-Score. Model accuracy is a metric that reflects the proportion of correct predictions made by a model and is calculated by the number of correct predictions divided by the total number of predictions made by the model. F1 score is a metric that combines both precision and recall, with higher scores indicating better performance. Precision is a measure of the accuracy of a model when it correctly predicts a positive outcome, while recall is a measure of the ability of a model to find all of the positive cases within the dataset. The area under the curve (AUC) measures the ability of a classifier to distinguish between positive and negative classes. It does this by comparing the true positive rate (TPR) against the false positive rate (FPR) at various classification thresholds. Like the F1 score, AUC ranges from 0 to 1, with a higher value indicating a better classifier^37^. We also present a full table of results for the logit model, along with confusion matrices for all models in the Supplementary Material.

In CT 1, the VGG-19 model achieves a test accuracy well above chance with a classification accuracy of 78% on the test set. The VGG-19 model can be considered an excellent discriminator between the two classes, with an AUC score of 0.8239 on the test set. Moving to the SVM, we observe a lower accuracy score (73%) and a lower AUC (0.7380). Logistic regression provides the worst performance with an accuracy of 67% and an AUC of 0.6796. When comparing the two image classification models, the VGG-19 achieves an F1-score of 0.7567 compared to 0.7265 from the SVM classifier. When analysing data from the test set during CT 2, we observe a similar pattern of results. We found that, in both cases, the test accuracy was well above chance, with the VGG-19 achieving an accuracy score of 65% and SVM scoring 61%, note that the weighted Average Area Under the Curve (wAUC) is reported for CT 2, where we can report wAUC scores of 0.7800 and 0.7114, respectively. Logistic regression falls short again with a performance of 0.5787 (wAUC) and an accuracy of 60%. Table 1 depicts the results for all the performance metrics for both CTs for VGG-19 and SVM. A full table of results for the logit model (Supplementary Tables 1–3) along with confusion matrices (Supplementary Figs. 3–8) for all models are reported in the Supplementary Material.

**Table 1:** Performance results of image classifiers on test set.

	VGG-19	VGG-19	SVM	SVM
	(CT 1)	(CT 2)	(CT 1)	(CT 2)
Accuracy	**0.783**	**0.648**	0.728	0.612
AUC	**0.824**	**0.78**	0.734	0.682
F1- Score*	**0.757**	**0.616**	0.727	0.575
**Percentage Accuracies**				
80% Percent	**0.716**	**0.648**	0.696	0.596
50% Percent	0.684	0.564	**0.704**	**0.592**
30% Percent	0.604	0.436	**0.608**	**0.476**
15% Percent	0.524	0.328	**0.576**	**0.376**
**Time Cut Off Accuracies**				
15 s	**0.776**	**0.64**	0.756	0.616
10 s	**0.728**	0.604	0.696	**0.620**
5 s	**0.684**	**0.544**	0.652	0.524
2 s	**0.608**	0.32	0.592	**0.384**

* For Classification Task 2, weighted average metrics are reported. Total number of test observations = 250. The best result of each trial is highlighted in bold.

**Figure 4:**
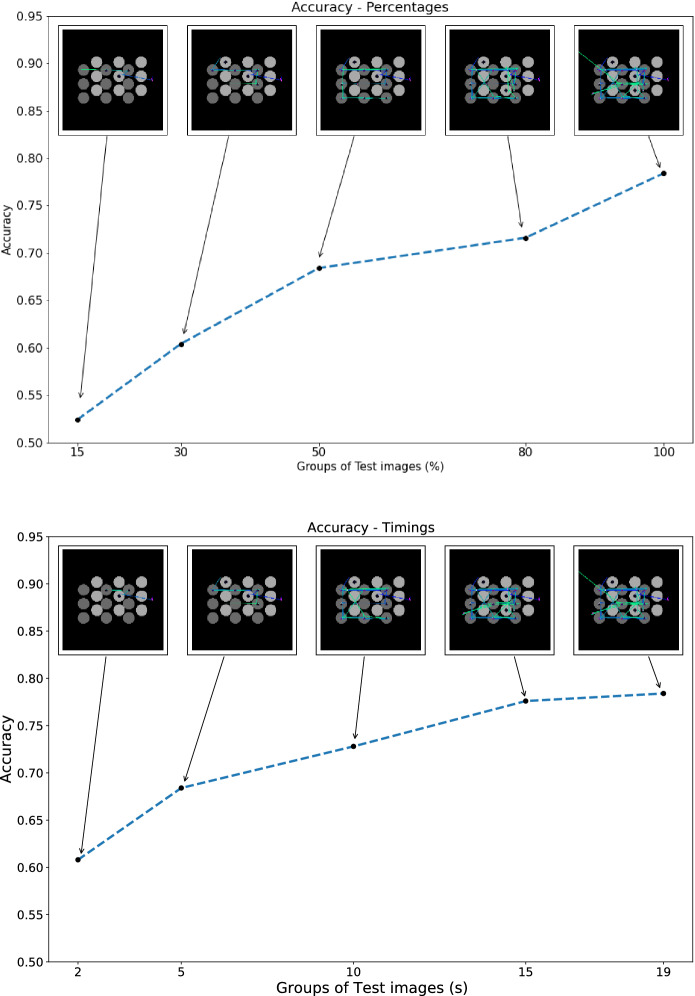
Accuracy of VGG-19 model in CT 1 using subsequences via percentages (**i**) and time points (**ii**).

When evaluating our trained models’ ability to classify partial scanpaths, we note the trend of a small decrease in model accuracy relative to the amount of data removed from the analysis, as illustrated for CT 1 in Fig. 4 below. Results for CT 2 are similar and reported in Supplementary Fig. 1 in the Supplementary Material. The results suggest that the gaze behaviour of different strategies forms distinguishable patterns early on in the visual search process despite the stochastic nature of inter-participant eye movements during game play. In CT 1, during model experiments using the sets of scanpaths created from a percentage of the total recorded sequence, we observe model accuracies at the following intervals: 80% interval (71% Accuracy, 0.7799 AUC), 50% interval (68% Accuracy, 0.7486 AUC), 30% interval (60% Accuracy, 0.6338 AUC), and the 15% interval (52% Accuracy, 0.4978 AUC). To put these findings into perspective, we observe a reduction of only 10% Accuracy when comparing the 50% interval to full scanpaths from the test set and perform better than chance from the 30% interval on-wards. During the experiments where arbitrary time points were formed, we observe a model accuracy of 72.8% with the VGG-19 model when using 10 s as the cutoff point, a reduction of close to 5%.

We are able to report a similar trend for the SVM classifier: 80% interval (69% Accuracy, 0.6925 AUC), 50% interval (70% Accuracy, 0.6882 AUC), 30% interval (60% Accuracy, 0.5576 AUC), 15% interval (58% Accuracy, 0.5074 AUC). We note that with very little data in both the 2 s trial and the 15 percent trial the SVM outperforms the VGG-19 model in terms of accuracy. While it is out of the scope of this paper, it remains an open question as to why this occurs.

We observed similar results during CT 2 where we found a 9% decrease in model accuracy when using the scanpaths created with 50% of the data and VGG-19 model. Due to the class imbalance, the small sample size of our data, and more nuanced differences between playing types, CT 2 is a much harder classification task for both models. During CT 2, when conducting the model experiments using the sets of scanpaths created from a percentage of the total recorded sequence, we observe model accuracies at the following intervals for the VGG-19 model: 80% interval (65% Accuracy, 0.7569 wAUC), 50% interval (56% Accuracy, 0.7141 wAUC), 30% interval (44% Accuracy, 0.6489 wAUC), and the 15% interval (33% Accuracy, 0.5406 wAUC). Again, we are able to report similar results of the SVM outperforming the VGG model when using shorter subsequences for the SVM observing model accuracy at the following intervals: 80% interval (59% Accuracy, 0.6714 wAUC), 50% interval (59% Accuracy, 0.6761 wAUC), 30% interval (48% Accuracy, 0.6069 wAUC), and the 15% interval (37% Accuracy, 0.5343 wAUC).

### Interpretation of model predictions

We aim to identify the features that contribute most significantly to the confidence of the model’s predictions in the two classification tasks (CT 1 and CT 2). To do so, we evaluate the relationship between the model’s Confidence Estimation (CE) and three features expressing the type and amount of information acquired: (1) the proportion of fixations on the other player’s payoffs, (2) the proportion of transitions between the other player’s payoffs, and (3) the natural logarithm of the response time as a proxy for the total amount of information acquired by the participant. Confidence estimation (CE) involves assessing the level of certainty that a deep learning model has in its own predictions, which is commonly achieved via computing a probability score^38^. We expect the CE to be directly linked to the characteristics of the visual analysis and only partially linked to the amount of information acquired (RT). In CT 1, we find that the CE of the model is positively correlated with the proportion of fixations on the other player’s payoffs (Pearson’s r = 0.76, *p*< 0.001) and with the proportion of transitions between the other player’s payoffs (Pearson’s r = 0.66, *p*< 0.001). We also find a significant, yet weaker, correlation between CE and response times (Pearson’s r = 0.31, *p*< 0.001).

In CT 2, we observe similar results when the model predicts a choice consistent with the equilibrium strategy: the CE of the model is positively correlated with both the proportion of transitions between the other player’s payoffs (Pearson’s r = 0.44, *p*< 0.001) and the proportion of fixations on the other player’s payoffs (Pearson’s r = 0.37, *p*< 0.001). However, we do not find a positive relationship between response times and CE in this case (Pearson’s r = −0.16, *p* = 0.06). When the model predicts a choice consistent with the Naïve strategy, we find that the proportion of transitions between the other player’s payoffs is negatively correlated with CE (Pearson’s r = −0.36, *p*< 0.001), while the proportion of transitions between the participant’s payoffs is positively correlated with CE (Pearson’s r = 0.40, *p*< 0.001). No relationship is observed between response times and CE in this case (Pearson’s r = −0.06, *p* = 0.56). Finally, we find that none of the features examined is correlated with CE when the model predicts a choice consistent with the Coordination strategy. This is in line with previous results showing that participants using a Coordination strategy devote the same amount of attention (expressed in terms of the proportion of fixations and transitions) to the incentives of the two players. Overall, our results are consistent with previous findings and suggest that the level of attention given to the incentives of the other player is a predictive factor in the decision-making strategy used^39^.

Finally, we test the hypothesis that the model makes better predictions in some games compared to other games and that the model is more accurate in predicting one strategy than another. We test the first hypothesis in CT 1 by running a mixed-effect logistic regression with accuracy of the model as dependent variable, game type (dominant solvable games and games that do not contain a dominant strategy) as independent variable, and Subject as random effect. Results of the model show no effects of the game type (B = 0.49, *p* = 0.12). We test the second hypothesis in CT 2 by running another mixed effect logistic regression analysis with the accuracy of the model as dependent variable, the type of strategy used by the participant (Equilibrium, Naïve, and Coordination) as independent variable, and Subject as random effect. Results show that the model is more efficient in predicting choices consistent with equilibrium strategy (Mean = 0.86) than choices consistent with Naïve (Mean = 0.67; B = −1.11, *p* = 0.004) or Coordination (Mean = 0.20; B = −3.24, *p*< 0.001) strategies. In particular, results show that the model underestimates the number of times participants choose in accordance with ”Coordination” strategy (only 5% of the time compared to 18% of choices observed). This may be due to the fact that the visual analysis required to implement the Coordination strategy can also be used to implement different strategies^16^.

## Discussion

In this work, we were able to successfully represent the cognitive process of participants’ playing economic games by transforming the recorded gaze data into scanpath images thus accounting for the spatiotemporal sequence within the data. This post-processing approach enables machine learning methods to accurately classify subsequences of the data from new participants using a model trained on only full sequences stemming from the training set. We were surprised by the large amount of data we could remove to form a subsequence and the resulting relatively small decrease in accuracy of prediction, further confirming that distinguishable patterns are formed very early on in the decision process^16,29^.

We focused our study on scanpath design showing its usefulness as model input by highlighting the results of both an out-of-the-box CNN and an SVM image classifier. Using scanpaths as model input both of our models outperform traditional methods to predict choice behavior from gaze data in games presented in normal form. Much like in previous studies we aimed to classify gaze behaviour indicative of players who select the Nash-equilibrium and separately, the exact strategy used by players in our games. We were able to anticipate choices accurately with very little data, with both models performing well over chance with only 30% of the data available in the scanpath subsequences, and with CT 2 containing a moderate class imbalance. Our findings support the well-established hypothesis that it is possible to classify participants by the depth of their strategic abilities using the pattern of their eye-movements^11,14,16,23^. Not only does our approach deliver a more accurate classification of strategic types, but also contributes to the literature by providing a method that could be developed in future experiments to create an adaptive counterpart that modifies its playing style based on the eye movements of the participant, opening the door to a plethora of new experimental designs in this domain.

Generating scanpaths from the data seems to come with certain advantages when analyzing games presented in normal form. In one of the most comparable studies^14^ the authors were unable to find a statistically significant relationship when investigating if they could identify the equilibrium choice in two by two games using saliency maps as input to a Salience Attentive Model (SAM). What makes this comparison so strong is that the games the authors used were created with the same experimental design as in our experiment. Our hypothesis as to why the authors of this study were unable to identify equilibrium choices using a saliency model is due to the loss of temporal information that occurs when creating saliency maps as fixations tend to be aggregated over the temporal dimension^40^. In a separate study^15^ a Multilayer Perceptron (MLP) was used with the following variables as input: reaction time, gaze dispersion and pupil deviation. The aim of the model was to detect whether a single trial is of the “predictable” or “unpredictable” type. The authors were able to classify these types with an accuracy of 67%, which is the most comparable analysis between the two papers. That being said, the two studies we compare our approach against were conducted in the context of two-by-two games which are characterized by a lower relational complexity of the payoff structure^31^, which may have effects on the classification difficulty of the patterns generated from recorded eye-movements.

When examining traditional modelling methods applied to players’ type classification, including cluster analysis and mixed model approaches do not reach the same level of accuracy observed with our approach^16,23,28,29,41^. Further, in these studies the accuracy of the models strongly depends on the strategic environment under consideration and the complexity of the decision rule used by the decision-maker. On average, in the contest of three-by-three games, the highest level of accuracy reported is around 68%^29^. Results from our baseline regression model are in line with these results achieving an accuracy of 67%, an 11% deficit when compared to our augmented VGG-19 model.

Much like in the field of vision research, experimental economists are searching for methods that can be used to predict across domains^11,42^. We are especially keen to use our scanpath design in different games to investigate the stability of levels of cognition across strategic environments of different types and complexity. Future research could simply attempt to classify scanpath images created from games with different structures using the fully trained model presented in this experiment, or build more complicated ML models to analyse the data. To date, limited research has been conducted which incorporates ML techniques, into economic games with a few notable exceptions^11,14,15^. We hope to continue to see developments of new analysis types in this field, as historically game paradigms have provided a much needed laboratory setting to study cognition in strategic environments which enables developments in theory that are then used to explain patterns in naturally occurring data^43,44^.

Outside of economic games, our method contributes towards the creation of interactive systems that incorporate gaze data^45^. Considerable resources have already been allocated to incorporating eye-tracking research into website design^46^, making the possibility of using a similar approach in more general domains by crafting AOIs into websites a priory quite a feasible task. We can envisage our approach being adapted to contexts such as shopping websites, online games played using virtual reality headsets, and in other strategic contexts where the cognitive state of the user is relevant such as stock trading, where this approach could be adapted to create a cooling off system to prevent traders from over-trading when losses start to occur.

Scanpath analysis is receiving increased recent attention due to the rise of Virtual and Augmented Reality systems^47^. While we made use of a VGG-19 pre-trained on the imagenet dataset largely for reasons of convenience, a number of deep learning techniques could be used to drastically improve the performance of our model^40^. Additionally, it remains an open question outside the scope of this paper as to why the SVM outperforms the VGG-19 model when minimal data is available. Outside of model selection, there are a few limitations to our study. First, our study suffers from a small sample size, for a study that uses ML methods. Second, there is a quite serious concern surrounding the generalizability of our experiment. It is widely accepted that task semantics greatly affect gaze behaviour^48^, it remains uncertain if our method of using subsequences to model cognition would achieve the same result in more noisy and complex environments outside of a laboratory setting^49^. While we are encouraged by results from previous studies^18,50^ which demonstrate the ability to classify subsequences of eye-tracking significantly above chance in a variety of settings, and in one instance using a similar kernel-based method, this remains an open question. Further, cognitive strategies in real-world settings will most probably be more nuanced and therefore harder to differentiate and classify using our approach.

From a policy perspective, our work demonstrates that gaze data can be used to provide an information advantage to AI-endowed players in strategic settings. As with all technology, it is not hard to imagine cases where eye-tracking data can be used to produce both good and bad outcomes for society. Trends such as the increase in the number of workers participating in the gig economy via smartphone apps^51^, the increased adoption of VR headsets, and the integration of eye-tracking data into online training and adaptive feedback systems, make it easy to envisage a world where eye-tracking data affect everyday life for a large percentage of the population. Our work here is just the tip of the iceberg so to speak, projects with a larger sample size could easily analyze gaze patterns at the participant level to customize user-based strategies. Much consideration and awareness of potential issues are needed going forward if eye-tracking data is to be successfully regulated as it becomes part of everyday life.

## Methods

### The games

To implement our ML classification method we used data from Marchiori et al.(2021)^19^. The authors conducted a behavioural experiment recording eye movements of participants playing a set of games characterized by a unique game theoretical optimal solution known as Nash Equilibrium^27^. In their study, the authors included three categories of games. In two of these categories, strategic sophistication is required in order to identify the Nash equilibrium. Strategic sophistication is the attempt to predict others’ decisions by taking their incentives into account^28,52^. In the third category, the equilibrium strategy can be identified without taking into account the other player incentives. In our experiment, we excluded games from this third category where the equilibrium strategy and more simple decision strategies overlap focusing on the two categories of games requiring strategic sophistication. Games in one selected category are called Dominant solvable Other (DO) games, and are characterized by the computer having a dominant strategy. This means that the computer has a strategy that provides strictly higher payoffs regardless of the strategy selected by the other player. Games in the second selected category are called Unique Equilibrium (UE) games, these games also include a unique Nash Equilibrium but do not contain a dominant strategy for either player and they are therefore not solvable by iterated dominance. Importantly, the Nash Equilibrium action and the actions corresponding with the Coordination and Naïve strategies were randomized across games to avoid regular patterns which could be recognised and exploited by a participant. See Supplementary Fig. S2 in the Supplementary material for the full list of games.

Previous eye-tracking research has shown that the analysis of the lookup patterns can be used to reconstruct, at the trial level, the decision process of different individuals and reveal the rationale behind their decisions^16^. In games, possible deviations from equilibrium depend mainly on three components: (i) the social preferences of the players, (ii) their strategic thinking ability, and (iii) their beliefs about the expected behavior of their counterparts. In their study, Marchiori et al.^19^ controlled for the first of these three components by providing information to the participants about the strategy of the computer. By telling the participants that the computer will play rationally with the aim of gaining as much as possible, they limited the participants’ propensity to cooperate by reducing their uncertainty towards the intentions and the strategic abilities of the counterpart. Possible deviations from equilibrium play are still possible and are mainly due to the inability of the player to properly represent the strategic environment, anticipate the other player’s behavior, and best respond to it.

### Analysis of information acquisition, strategic play

In our games, equilibrium play requires forming beliefs about the other player’s actions. In terms of information acquisition, the belief formation process occurs through the use of saccades between the payoffs of the counterpart. Saccades express rapid eye movements connecting consecutive fixations and can be viewed as a valid and reliable measure of how individuals acquire and integrate different pieces of information^23,29^. In the two categories of games selected for our experiment, looking at the other player’s payoffs is not sufficient to identify the optimal solution of a game. Previous results show that equilibrium play, in games that require strategic sophistication, is associated with a large use of vertical and horizontal saccades between the other player’s payoffs which are necessary to form accurate beliefs about the possible action of the counterpart, and vertical saccades between player’s own payoffs which are necessary to identify the best response to it^16^. Conversely, the implementation of a Naïve strategy is characterized by a high proportion of horizontal saccades between the payoffs of the player and more generally a high proportion of fixations on the player’s own payoffs. Finally, actions consistent with a coordination strategy are usually associated with a higher proportion of diagonal saccades between the payoffs of the two players for each possible outcome of the game and in general balanced attention between own and other payoffs. Given their relevance for the identification of the decision strategy used by the individual, information about saccades are made salient via the use of colour when creating the scanpaths.

### Experimental procedure

We use data from the entire pool of participants (243) who took part in the assessment stage of Marchiori et al.(2021)^19^ experiment (81 males, mean age = 24.1, SD age = 4.6). In the assessment stage, the authors assessed the initial level of strategic sophistication of each participant, and no feedback was provided to them. During this initial phase, each participant played five DO games and five UE games for a total of 2430 games played.

They played a set of two-person 3x3 games presented in normal form against a computer programmed to always play the action consistent with the equilibrium strategy. Participants were informed that the computer would always play rationally and try to maximize its own payoff. To facilitate comprehension, the payoffs of the two players in the game matrix were presented in different colours (see Fig. 1). Participants were not subject to a time constraint during the experimental task. At the end of the experiment, three trials were randomly chosen, and the participant was paid based on the outcome of the selected games. The experiment was conducted at the Experimental Psychology Laboratory of the University of Trento (Italy). The assessment stage of the experiment lasted about 15 minutes. The entire experiment lasted about 1 hour. The study was approved by the Human Research Ethics Committee of the University of Trento (protocol title: “Transfer learning within and between brains”). All participants gave informed consent. All experiments were performed in accordance with the relevant guidelines and regulations. Informed consent was obtained from all participants before the start of the experiment.

### Eye-tracking recording

Eye movements were monitored and recorded using an Eyelink 1000 tower mount (SR research, Ontario, Canada) at a sampling rate of 1000 HZ. The authors used a custom-made calibration procedure with 13 points. Points were placed at the center of the nine cells and in the four possible locations of the fixation cross. The point located at the center of the matrix (corresponding to the central cell of the matrix) was repeated twice. After the calibration phase, a validation phase was performed to test the accuracy of the calibration. Calibration was repeated if necessary. During the experimental task, and before the beginning of each trial, a drift correction was performed in order to test whether participants looked at the current fixation location, by means of a target stimulus that was randomly presented in one of four possible locations. The matrix game was presented to the participant after the target stimulus was fixated for 300 ms. This was done to ensure that each participant, in every trial, was looking at one of four possible random points located outside the matrix before starting the game. To transform the data into scanpaths, the data was processed using the PyGaze library^53^, with colour maps from Matplotlib^32^.

### Model architectures and training procedure

We implemented a transfer learning and fine-tuning strategy using a VGG-19 model pre-trained on Imagenet as the baseline model. In both Classification Tasks, we freeze the convolutional base layers and replace the fully connected layers and final classification layer of the model with a custom head. We used Stochastic Gradient Descent (SDG) as the optimiser. Initially, we implemented Bayesian hyperparameter tuning via the Keras tuner^54^ to select: (i) the number of fully connected layers, (ii) the number of nodes in each fully connected layer, and (iii) the dropout percentage between these layers. To improve classification accuracy, we fined-tuned the model by unfreezing some of the convolutional layers of the model and lowering the learning rate. To choose the optimal parameters during this stage, we used a random grid search which adjusts the number of frozen layers and the learning rate at each iteration.

As the target dataset differs greatly from the source dataset a number of regularization steps were also included in the model to help boost model performance and prevent overfitting. First, a Global Average Pooling (GAP) layer was inserted after the convolutional layers of the model to be used as a structure regularizer^55^. Additional measures include a light amount of L1/L2 regularization *(1e-5)* which is applied to the model alongside an early stopping callback. For both CTs, the model was able to train for a maximum of thirty epochs and up to ten further epochs during the fine-tuning stage.

In CT 2, to deal with the class imbalance problem, we implemented a focal loss function with the default values for the alpha and gamma parameters. The focal loss was designed to deal with class imbalance by down-weighting well-classified examples and focusing on harder examples^56^. All models were implemented using Tensorflow-Keras library^57^, which makes the Imagenet weights available for the VGG-19 model from its applications module, hence reducing the computational cost and time of running our experiments and making our experiments easier to reproduce.

### Comparison with baseline models

We compared our model to two common approaches used for classification problems, namely, a Support Vector Machine (SVM) image classifier and a logistic regression model. In the cases of the SVM, we implemented a Radial-Basis-Function (RBF) kernel SVM with the SVC estimator from the Python SkLearn library^37^. We used the same scanpaths as model input. We apply a 5-fold cross-validated, exhaustive parameter grid search to select the values of the hyperparameters. We set the kernel coefficient and regularization penalty to 0.0001 and 10, respectively, in the exact Machine Learning strategy task, the penalty parameter was computed with adjusted weights inversely proportional to class frequencies in the input data. To fit the logistic regression model to CT 1, we used a generalized linear model (GLM) from the R package stats (version 3.6.0). For CT 2, we applied a multinomial regression model within the R package nnet (version 7.3.16)^58^. We fitted different models by regressing the binary outcome on proportions of *‘Own’, ‘Other’ and ‘Intracell’* transitions recorded in intervals of the total duration of the game (at 15%, 30%, 50%, 80%, 100%) or in fixed time spans of 2, 5, 10 and 15 s.

## Supplementary Information

Supplementary Information.

## Data Availability

All datasets used in the current study and the scanpaths generated are available in a dedicated OSF repository at DOI https://doi.org/10.17605/OSF.IO/HJUPE. All code to recreate the models is available at https://github.com/seanbyrne226/Choice-Behaviour-Machine-Learning-Modeling-of-Scanpath-Subsequences.
