# Supplementary material for: Predicting choice behaviour in economic games using gaze data encoded as scanpath images
Source: Sci Rep. 2023 Mar 23;13:4722. doi: 10.1038/s41598-023-31536-5 (PMC10036613; doi:10.1038/s41598-023-31536-5)
Supplement: Supplementary file 1 — Supplementary Information. [file 41598_2023_31536_MOESM1_ESM.pdf]

Supplementary material

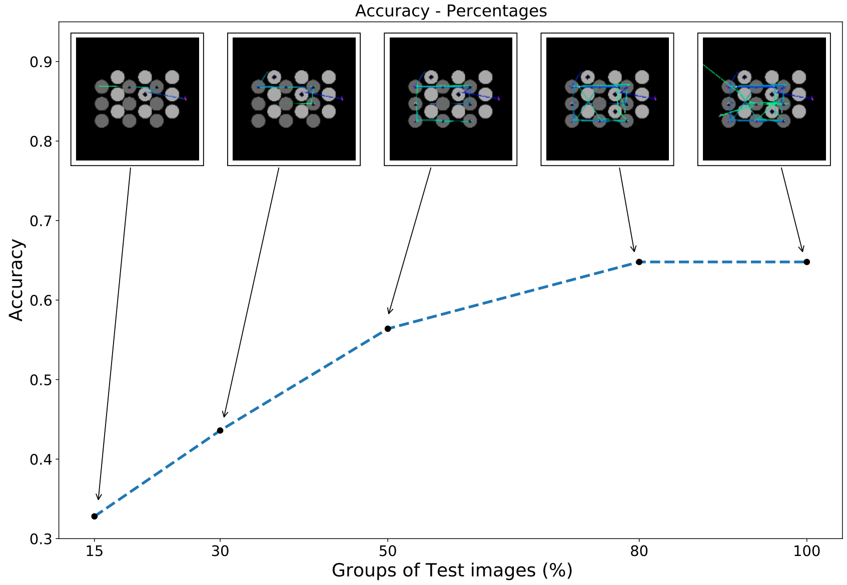

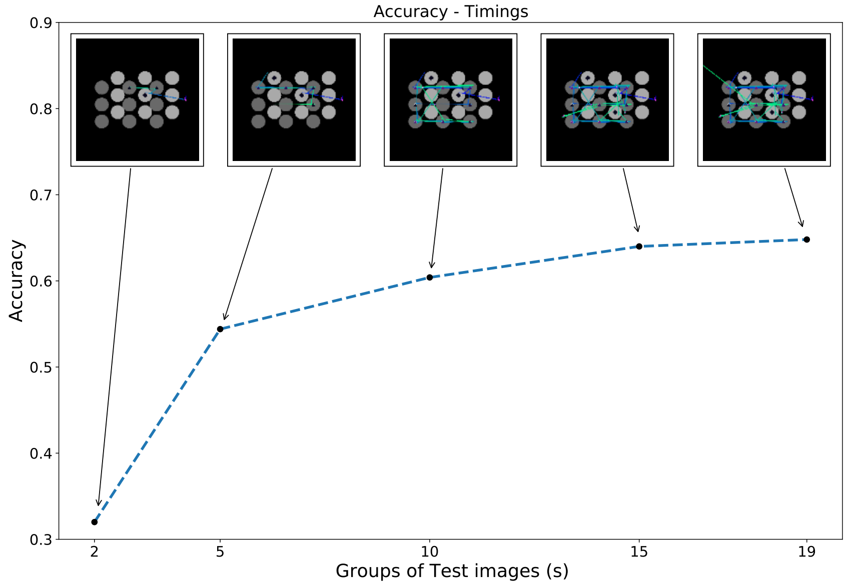

Supplementary Figure 1. Accuracy of VGG-19 model in CT 2 using subsequences via percentages (1i) and time points (1ii).

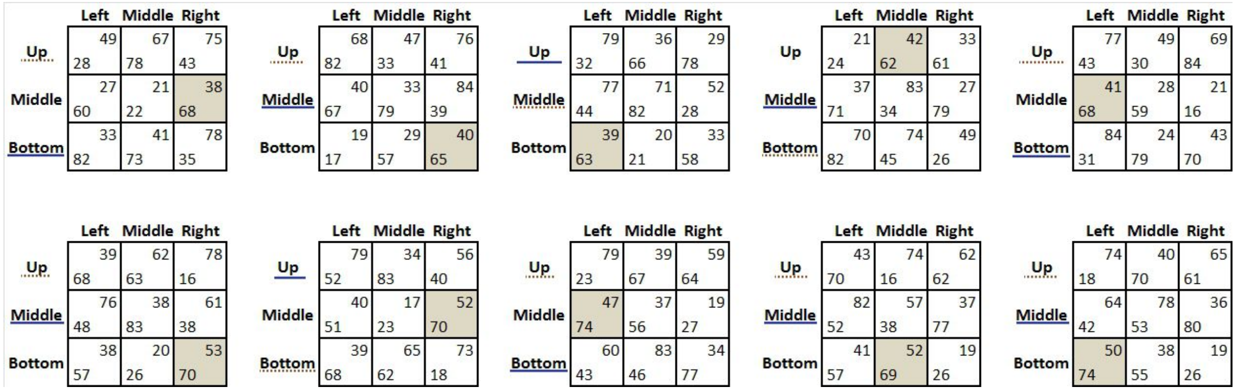

Supplementary Figure 2. The games used in the experiment grouped by types. The payoffs of the Row/Column player are located in the Bottom-left/Upper-right part of the nine cells. The Nash equilibrium payoffs is indicated in grey. The Naive strategy is underlined with a solid line and the Coordination strategy with a dashed line.

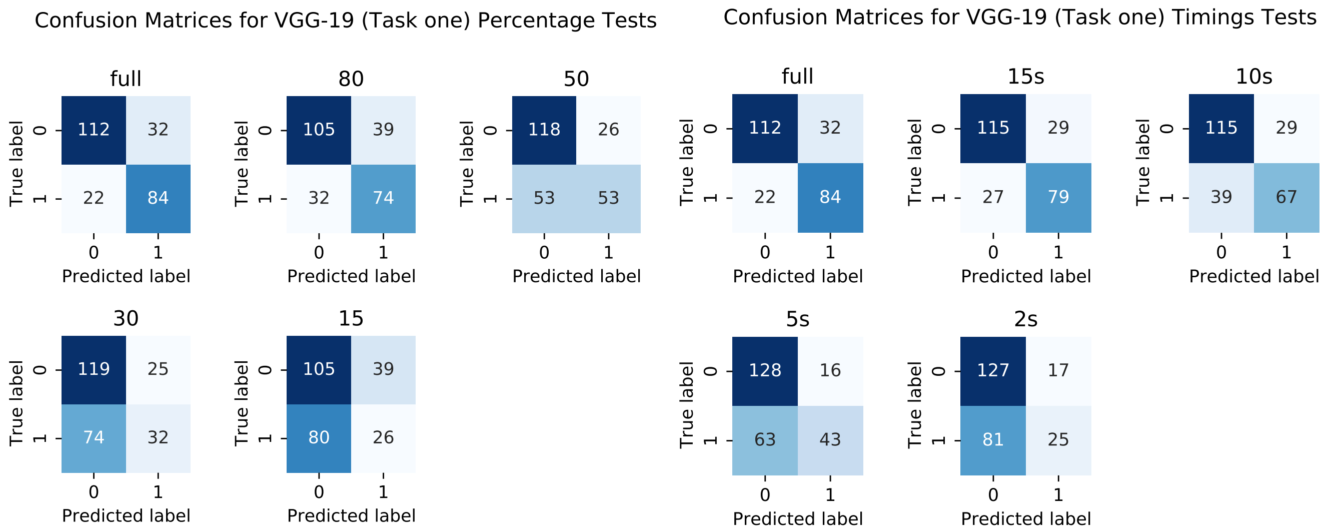

(i) Percentages

(ii) Timings

Supplementary Figure 3. Confusion Matrices of VGG19-model in CT 1 using subsequences via percentages (3i) and time points (3ii)

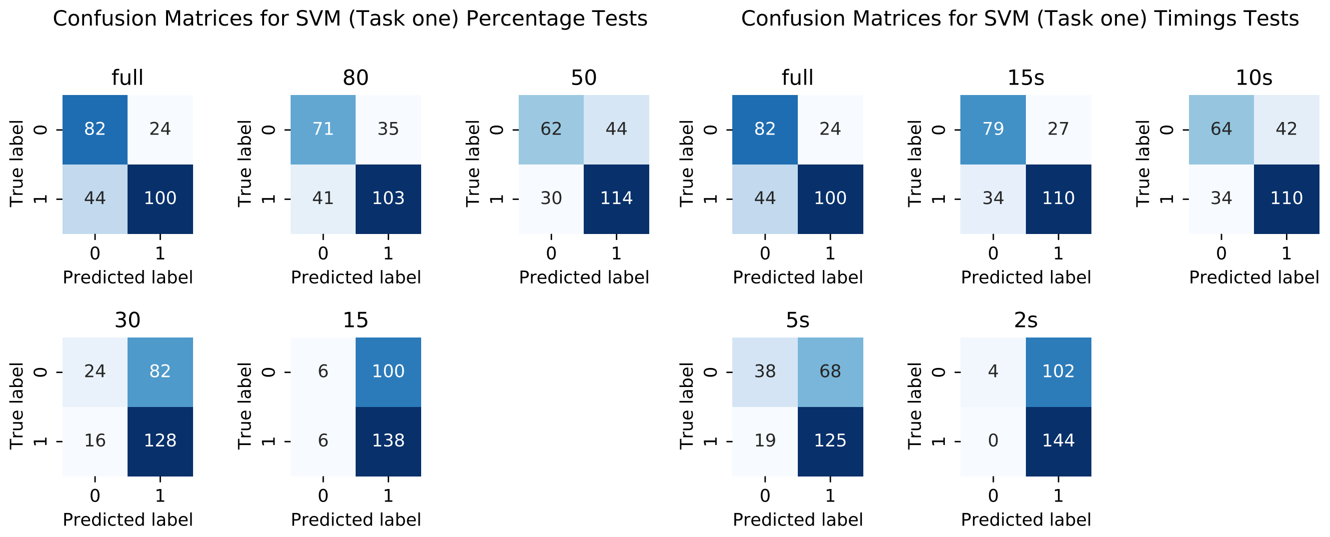

(i) Percentages **(ii)** Timings

Supplementary Figure 4. Confusion Matrices of SVM-model in CT 1 using subsequences via percentages (4i) and time points (4ii)

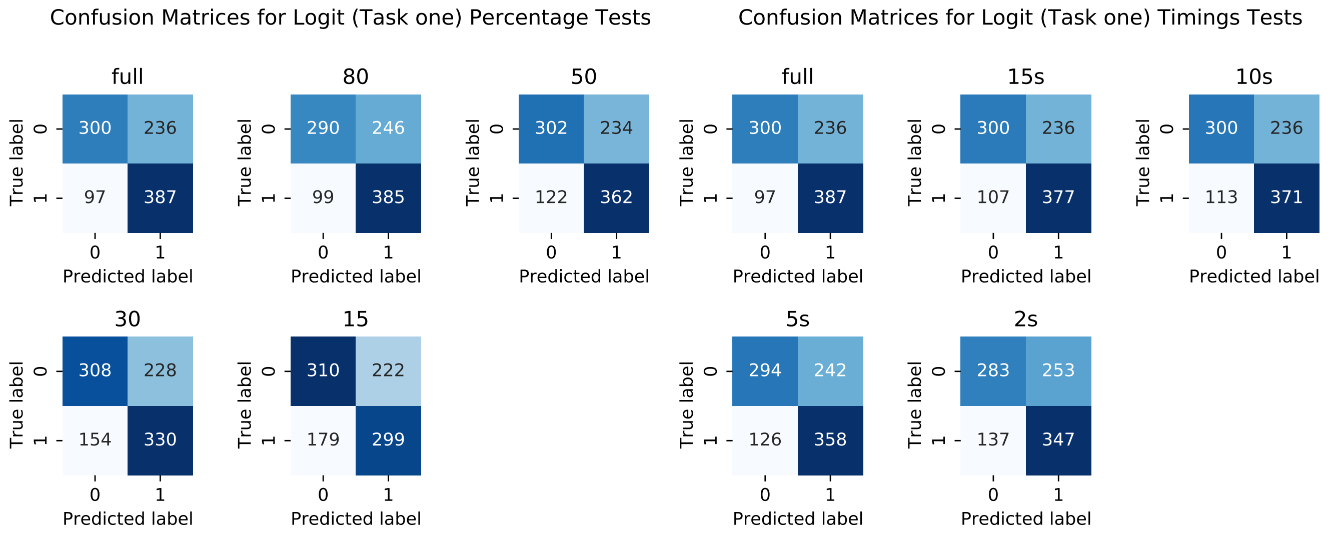

(i) Percentages **(ii)** Timings

Supplementary Figure 5. Confusion Matrices of Logit regression model in CT 1 using subsequences via percentages (5i) and time points (5ii)

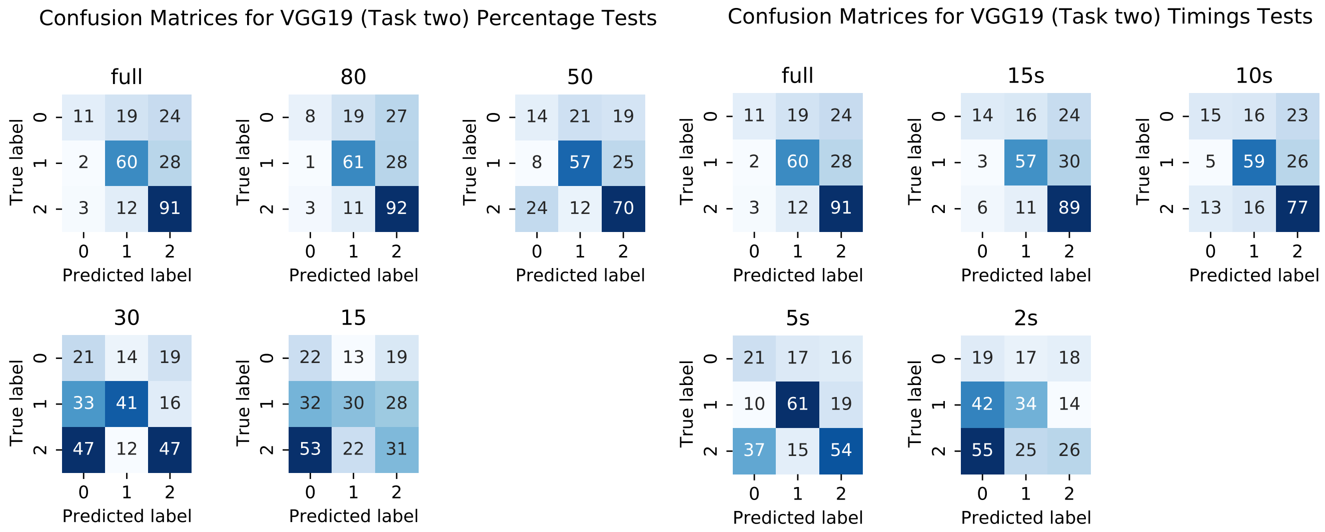

(i) Percentages **(ii)** Timings

Supplementary Figure 6. Confusion Matrices of VGG19-model in CT 2 using subsequences via percentages (6i) and time points (6ii)

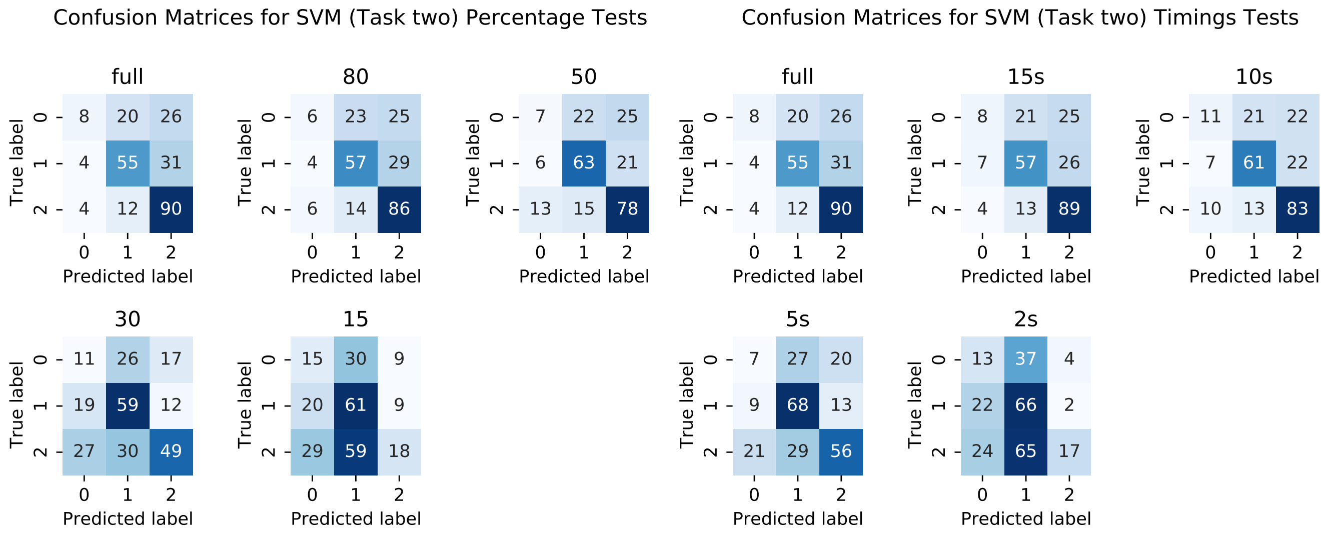

(i) Percentages **(ii)** Timings

Supplementary Figure 7. Confusion Matrices of SVM-model in CT 2 using subsequences via percentages (7i) and time points (7ii)

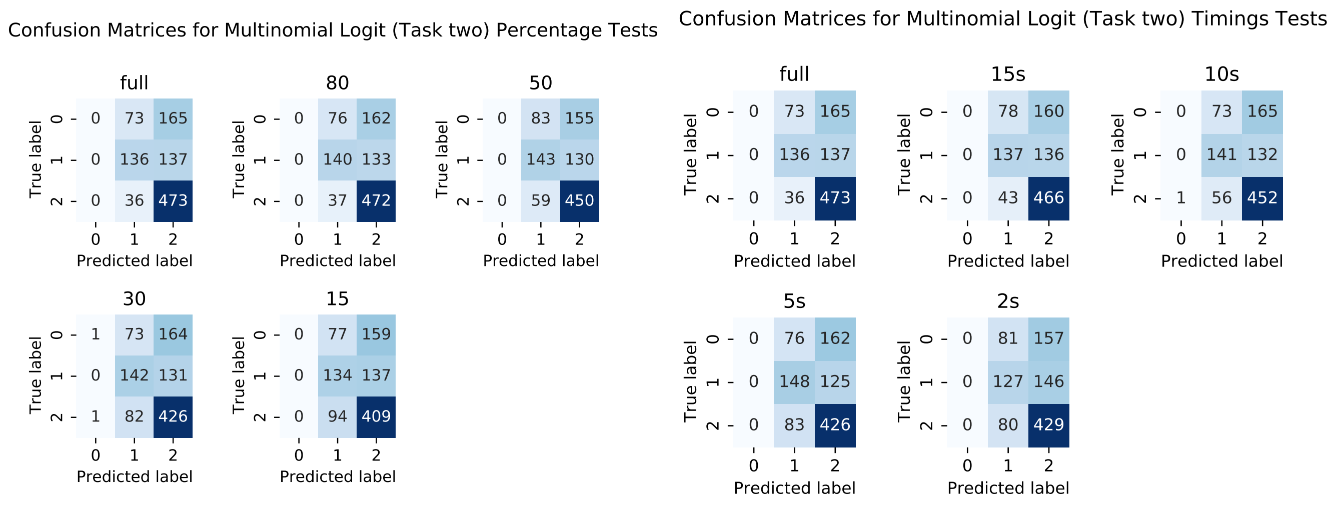

(i) Percentages

(ii) Timings

Supplementary Figure 8. Confusion Matrices of Multinomial Logit regression model in CT 2 using subsequences via percentages (8i) and time points (8ii)

Percentages	Variable	Estimate	Std. Error	z value	Pr(>|z|)
Full	(Intercept)	0.6289	0.2918	2.16	0.0312
	Own	-4.2517	0.5469	-7.77	0.0000
	Other	2.8274	0.5590	5.06	0.0000
	Intracell	-3.0616	1.4590	-2.10	0.0359
80	(Intercept)	0.6058	0.2737	2.21	0.0269
	Own	-4.1175	0.5168	-7.97	0.0000
	Other	2.1976	0.5102	4.31	0.0000
	Intracell	-2.1822	1.3381	-1.63	0.1029
50	(Intercept)	0.5141	0.2426	2.12	0.0340
	Own	-3.4802	0.4586	-7.59	0.0000
	Other	1.4195	0.4386	3.24	0.0012
	Intracell	-0.9786	1.1078	-0.88	0.3770
30	(Intercept)	0.3457	0.1958	1.77	0.0774
	Own	-2.5292	0.3724	-6.79	0.0000
	Other	1.3784	0.3724	3.70	0.0002
	Intracell	-1.2306	0.7986	-1.54	0.1233
15	(Intercept)	0.1362	0.1475	0.92	0.3557
	Own	-1.7372	0.2930	-5.93	0.0000
	Other	1.4423	0.3140	4.59	0.0000
	Intracell	-0.5133	0.4307	-1.19	0.2334

Timings	Variable	Estimate	Std. Error	z value	Pr(>|z|)
Full	(Intercept)	0.6289	0.2918	2.16	0.0312
	Own	-4.2517	0.5469	-7.77	0.0000
	Other	2.8274	0.5590	5.06	0.0000
	Intracell	-3.0616	1.4590	-2.10	0.0359
15s	(Intercept)	0.5912	0.2785	2.12	0.0338
	Own	-3.9147	0.5151	-7.60	0.0000
	Other	2.3470	0.5233	4.49	0.0000
	Intracell	-2.3422	1.3380	-1.75	0.0800
10s	(Intercept)	0.6270	0.2614	2.40	0.0164
	Own	-3.8105	0.4868	-7.83	0.0000
	Other	1.8515	0.4853	3.81	0.0001
	Intracell	-1.7461	1.2094	-1.44	0.1488
5s	(Intercept)	0.6157	0.2363	2.61	0.0092
	Own	-3.3889	0.4371	-7.75	0.0000
	Other	1.1590	0.4334	2.67	0.0075
	Intracell	-0.7870	0.9360	-0.84	0.4005
2s	(Intercept)	0.4281	0.1781	2.40	0.0162
	Own	-2.5519	0.3409	-7.48	0.0000
	Other	0.6410	0.3451	1.86	0.0633
	Intracell	0.2406	0.5172	0.47	0.6418

Supplementary Table 1. Logistic *(Logit)* Regression Estimates for Task 1.

**Percentages**	Variable	Estimate	Std. Error	Pr(>|z|)	z value
Full	Intracell (Naive)	-3.01	1.92	0.12	-1.57
	Other (Naive)	-4.40	0.86	0.00	-5.10
	Own (Naive)	1.28	0.66	0.05	1.94
	Intercept (Naive)	0.68	0.39	0.08	1.72
	Intracell (NE)	-3.07	1.65	0.06	-1.86
	Other (NE)	1.18	0.67	0.08	1.77
	Own (NE)	-2.97	0.64	0.00	-4.64
	Intercept (NE)	1.51	0.35	0.00	4.35
80	Intracell (Naive)	-1.39	1.79	0.44	-0.78
	Other (Naive)	-3.63	0.79	0.00	-4.59
	Own (Naive)	1.47	0.62	0.02	2.37
	Intercept (Naive)	0.45	0.37	0.22	1.22
	Intracell (NE)	-1.75	1.56	0.26	-1.12
	Other (NE)	0.95	0.61	0.12	1.56
	Own (NE)	-2.81	0.61	0.00	-4.60
	Intercept (NE)	1.40	0.33	0.00	4.26
50	Intracell (Naive)	-0.96	1.53	0.53	-0.63
	Other (Naive)	-3.01	0.69	0.00	-4.34
	Own (Naive)	1.29	0.56	0.02	2.29
	Intercept (Naive)	0.41	0.33	0.22	1.23
	Intracell (NE)	-0.96	1.35	0.48	-0.71
	Other (NE)	0.45	0.53	0.39	0.86
	Own (NE)	-2.43	0.55	0.00	-4.39
	Intercept (NE)	1.37	0.30	0.00	4.61
30	Intracell (Naive)	-1.60	1.08	0.14	-1.48
	Other (Naive)	-3.09	0.59	0.00	-5.21
	Own (Naive)	0.48	0.46	0.29	1.05
	Intercept (Naive)	0.73	0.27	0.01	2.70
	Intracell (NE)	-1.83	0.97	0.06	-1.89
	Other (NE)	0.09	0.45	0.85	0.19
	Own (NE)	-2.09	0.46	0.00	-4.55
	Intercept (NE)	1.48	0.25	0.00	5.96
15	Intracell (Naive)	-0.57	0.61	0.35	-0.94
	Other (Naive)	-2.67	0.52	0.00	-5.12
	Own (Naive)	0.42	0.37	0.27	1.11
	Intercept (Naive)	0.63	0.21	0.00	2.95
	Intracell (NE)	-0.56	0.54	0.30	-1.03
	Other (NE)	0.28	0.38	0.47	0.72
	Own (NE)	-1.47	0.37	0.00	-3.95
	Intercept (NE)	1.25	0.19	0.00	6.47

Supplementary Table 2. Multinomial Regression Estimates for Task 2 (percentages).

Timings	Variable	Estimate	Std. Error	Pr(>|z|)	z value
Full	Intracell (Naive)	-3.01	1.92	0.12	-1.57
	Other (Naive)	-4.40	0.86	0.00	-5.10
	Own (Naive)	1.28	0.66	0.05	1.94
	Intercept (Naive)	0.68	0.39	0.08	1.72
	Intracell (NE)	-3.07	1.65	0.06	-1.86
	Other (NE)	1.18	0.67	0.08	1.77
	Own (NE)	-2.97	0.64	0.00	-4.64
	Intercept (NE)	1.51	0.35	0.00	4.35
15s	Intracell (Naive)	-1.88	1.80	0.30	-1.05
	Other (Naive)	-3.53	0.80	0.00	-4.39
	Own (Naive)	1.36	0.64	0.03	2.13
	Intercept (Naive)	0.47	0.38	0.21	1.24
	Intracell (NE)	-2.38	1.56	0.13	-1.53
	Other (NE)	1.00	0.63	0.11	1.58
	Own (NE)	-2.77	0.61	0.00	-4.52
	Intercept (NE)	1.47	0.34	0.00	4.34
10s	Intracell (Naive)	-1.55	1.68	0.35	-0.93
	Other (Naive)	-3.07	0.76	0.00	-4.06
	Own (Naive)	1.39	0.62	0.02	2.26
	Intercept (Naive)	0.38	0.37	0.30	1.04
	Intracell (NE)	-1.81	1.44	0.21	-1.25
	Other (NE)	0.67	0.59	0.25	1.14
	Own (NE)	-2.70	0.59	0.00	-4.61
	Intercept (NE)	1.49	0.32	0.00	4.64
5s	Intracell (Naive)	-1.79	1.36	0.19	-1.32
	Other (Naive)	-2.95	0.69	0.00	-4.29
	Own (Naive)	1.00	0.57	0.08	1.77
	Intercept (Naive)	0.50	0.34	0.14	1.48
	Intracell (NE)	-1.45	1.13	0.20	-1.29
	Other (NE)	-0.01	0.53	0.98	-0.02
	Own (NE)	-2.58	0.53	0.00	-4.83
	Intercept (NE)	1.60	0.29	0.00	5.44
2s	Intracell (Naive)	-1.73	0.80	0.03	-2.18
	Other (Naive)	-3.03	0.57	0.00	-5.36
	Own (Naive)	0.18	0.44	0.68	0.41
	Intercept (Naive)	0.85	0.26	0.00	3.25
	Intracell (NE)	-0.36	0.66	0.58	-0.55
	Other (NE)	-0.55	0.43	0.20	-1.29
	Own (NE)	-2.30	0.43	0.00	-5.35
	Intercept (NE)	1.60	0.23	0.00	6.86

Supplementary Table 3. Multinomial Regression Estimates for Task 2 (timings).

Intra-individual consistency in decision strategies across games

Level of consistency	100%	90%	80%	70%	Total
Proportion of Subjects	14%	11%	13%	14%	51%
Number of Subjects	34	26	31	33	124

Supplementary Table 4. Proportion and number of participants who use the same strategy 100%, 90%, 80% and 70% of the time over the course of the ten games.
